# Teamwork to Survive in Hostile Soils: Use of Plant Growth-Promoting Bacteria to Ameliorate Soil Salinity Stress in Crops

**DOI:** 10.3390/microorganisms10010150

**Published:** 2022-01-12

**Authors:** Rafael Jiménez-Mejía, Ricardo I. Medina-Estrada, Santos Carballar-Hernández, Ma. del Carmen Orozco-Mosqueda, Gustavo Santoyo, Pedro D. Loeza-Lara

**Affiliations:** 1 Licenciatura en Genómica Alimentaria, Universidad de La Ciénega del Estado de Michoacán de Ocampo (UCEMICH), Sahuayo 59103, Mexico; rjimenez@ucemich.edu.mx (R.J.-M.); rimedina@ucemich.edu.mx (R.I.M.-E.); scarballar@ucemich.edu.mx (S.C.-H.); 2 Facultad de Agrobiología “Presidente Juárez”, Universidad Michoacana de San Nicolás de Hidalgo (UMSNH), Uruapan 60170, Mexico; carmen.orozco@umich.mx; 3 Instituto de Investigaciones Químico Biológicas, Universidad Michoacana de San Nicolás de Hidalgo (UMSNH), Morelia 58030, Mexico; gustavo.santoyo@umich.mx

**Keywords:** plant growth-promoting bacteria, abiotic stresses, salinity, tolerance, biofertilizer, sustainable agriculture

## Abstract

Plants and their microbiomes, including plant growth-promoting bacteria (PGPB), can work as a team to reduce the adverse effects of different types of stress, including drought, heat, cold, and heavy metals stresses, as well as salinity in soils. These abiotic stresses are reviewed here, with an emphasis on salinity and its negative consequences on crops, due to their wide presence in cultivable soils around the world. Likewise, the factors that stimulate the salinity of soils and their impact on microbial diversity and plant physiology were also analyzed. In addition, the saline soils that exist in Mexico were analyzed as a case study. We also made some proposals for a more extensive use of bacterial bioinoculants in agriculture, particularly in developing countries. Finally, PGPB are highly relevant and extremely helpful in counteracting the toxic effects of soil salinity and improving crop growth and production; therefore, their use should be intensively promoted.

## 1. Introduction

One of the main challenges for agriculture globally will be to fulfill increasing food demand for a growing population, as it is estimated that by 2050, it will reach >9 billion, which indicates an urgent need to elevate agricultural production over the following decades [1,2]. However, plant growth, productivity, yield, and food quality are severely affected by biotic and abiotic stresses [3]. The first includes damage caused by several pests and pathogens, while the second includes drought, temperature, heavy metals, and salinity [4].

Soil salinity stress is considered to be highly detrimental for agriculture because of its devastating effects on productivity and food security, in addition to having important ecological and socio-economic repercussions [5]. It has been estimated that by 2050, approximately 50% of the global agricultural land will be affected by some level of salinity because of its constant rise [6]. The salinity of arable soil is primarily promoted by the accumulation of sodium (Na^+^) and chloride (Cl^−^) ions. Several factors contribute to the deposition of these salts, including those of natural and human origin, such as high evaporation rates and agricultural irrigation [7,8].

The accumulation of salts in soil limits water conductance, as well as soil porosity and aeration [9]. Similarly, salinity negatively affects microbial diversity in the plant rhizosphere [10]. Likewise, plants that grow in saline soil undergo morphological, physiological, and molecular changes that restrict growth and development [4]. Salinity also affects enzymatic activities, stomatal function, and photosynthetic rates, and increases the synthesis of reactive oxygen species (ROS), which damage cell membranes, lipids, proteins, DNA, and RNA, and induces programmed cell death [8,11]. Finally, this abiotic factor promotes hypertonic stress due to the accumulation of Na^+^ and Cl^−^ ions [4].

Several studies have shown that plants have evolved mechanisms to tolerate salts, such as antioxidant enzyme activation, ion homeostasis, polyamine synthesis, compatible solutes, osmoprotectants, and hormone modulation [12]. Nevertheless, microorganisms can improve soil physicochemical characteristics and help plants resist adverse environmental conditions [2,13,14]. The rhizosphere harbors a complex microbial diversity; however, some plant growth-promoting bacteria (PGPB) are distinguished, as, in addition to promoting plant growth, they stimulate their tolerance to saline stress [2].

PGPB establish favorable ecological relationships with plants, stimulating their growth through direct and indirect mechanisms. The former includes biological nitrogen fixation, synthesis of organic acids and siderophores, modulation of phytohormone synthesis, and activity of the aminocyclopropane-1-carboxylic acid deaminase enzyme. The indirect mechanisms include growth inhibition of phytopathogens through competition for space and nutrients, antibiosis by secondary metabolites, volatile organic compounds, and lytic enzymes, induction of plant immune responses, and the improvement of soil physicochemical properties [15,16]. During salinity stress, PGPB can promote nutrient uptake and homeostasis, as well as increase antioxidant activity, while promoting plant growth [4].

Salinity is one of the main threats to agricultural soils, which affects more than 100 countries in all climatic regions and has different costs and characteristics [17]. In Mexico, salinity occurs in arid and semi-arid regions (north and center of the country), mainly where agricultural irrigation is used, and soils have poor drainage and high evaporation. It has been reported that the affected area involves around 1 million hectares, which is critical because the productivity of crops decreases significantly with increasing salinity.

This issue can be addressed with the use of PGPB, which will have a positive impact on the recovery of soil fertility, as well as help plants tolerate salt stress. Additionally, the use of PGPB as a biofertilizer is a strategy that promotes sustainable agriculture, as they do not exhibit negative effects on the environment and human and animal health, and therefore are ecosystem-friendly [16]. However, to achieve this, it is necessary to understand the mechanisms by which PGPB allow plants to resist soil salinity stress. This will allow the selection of the best PGPB to produce efficient bioinoculants for agricultural crops.

## 2. Abiotic Stresses Effects on Agricultural Crops

Many agricultural crops grow in unsuitable environments, which do not allow plants to exploit their genetic potential for growth, development, and production [18]. This causes crop losses and can be explained by the effect of unfavorable environmental and growth conditions of a particular crop. When these conditions cause potentially harmful physiological changes in plants, this is referred to as a stressor effect [19,20]. Biotic stress includes damage caused by pests and pathogens, including fungi, bacteria, viruses, nematodes, and herbivorous insects, while abiotic stress includes heat, cold, drought, heavy metal contamination, and soil salinity. These factors have been widely reported to limit crop production in different proportions [21]. Despite the difficulty in calculating the effects of abiotic stress, some authors have indicated that approximately 96.5% of rural agricultural crops are affected by abiotic factors [22].

### 2.1. Heat Stress Effects in Crops

Temperature plays a fundamental role in growth and crop development, as it regulates the cellular metabolism of plants, and therefore an increase above the optimal level is registered as an environmental stressor [23]. Heat stress is defined as an elevation in temperature above a certain level and in exposure time, damaging plants. This type of stress can modify cell homeostasis, which results in negative effects on plant physiology, thus representing a significant risk to global agricultural production [24]. Specifically, an increase in temperature delays the germination of seeds, which promotes changes in crop planting density; otherwise, it limits the life cycle of certain crops, which induces senescence and shortens the growing season. Likewise, cereals such as rice (*Oryza sativa*), wheat (*Triticum aestivum*), and corn (*Zea mays*) can tolerate narrow ranges of temperature and modifications can cause seed damage, hence reducing yields [25].

### 2.2. Cold Stress Effects in Crops

Cold stress is another environmental factor that greatly limits growth and plant development, mainly in tropical and subtropical regions, and involves crops such as tomato (*Solanum lycopersicum*), *O. sativa*, *Z. mays*, and fruits, such as papaya (*Carica papaya*), banana (*Musa* × *paradisiaca*), and mango (*Manguifera indica*) [24]. This type of environmental stress is classified as chilling stress (0–15 °C) and freezing stress (<0 °C), which alters plant structure and metabolism, including those involved in photosynthetic and respiratory processes. Likewise, increased intracellular Ca^+^ and reactive oxygen species (ROS) accumulation takes place, with a decrease in membrane fluidity. In addition, low temperatures can cause drastic dehydration due to a cell’s inability to take water and ice formation, which causes protein denaturation. Depending on the severity and exposure time, cold stress can cause, among others, the appearance of superficial lesions in plant leaves, discoloration or yellowing, senescence, wilting, and rotting [24,26,27,28]. In the same way, cold can damage the propagative organs of crops, which affects seed production and the yield obtained [28].

### 2.3. Drought Stress Effects in Crops

Drought is also one of the main types of environmental stress that negatively influences agricultural crop yield worldwide. Between 80% and 95% of plant fresh biomass is made up of water, and consequently, this molecule is important in several physiological processes such as growth, development, and metabolism [29]. At present, effects of drought on agriculture are aggravated due to rainfall variability, stimulated by climate change, as well as a declining water supply, growing demand for the liquid due to population increase, accelerated evapotranspiration, and water retention capacity in the rhizosphere, among others [24,30]. Due to this, different physiological and morphological plant processes are affected by drought stress, which impacts both yield and crop quality [31]. Drought effects include defective seed germination, decreased growth and crop development, reduced plant nutrient availability, diminished photosynthesis, and low fresh and dry weights [24,32].

### 2.4. Heavy Metals Stress Effects in Crops

Various anthropogenic activities, such as the excessive use of inorganic chemical fertilizers in agriculture, urbanization, improper disposal of industrial and automobile waste, and wastewater, have allowed the accumulation of toxic metals such as copper (Cu), nickel (Ni), manganese (Mn), mercury (Hg), cadmium (Cd), copper (Co), iron (Fe), chromium (Cr), and zinc (Zn), in groundwater sources, or on surface soil [33]. Some of these metals are not useful to plants and cause stress by reducing their growth due to a decreased photosynthetic rate, poor nutrition, and reduced essential enzyme activity. They also interfere with membrane integrity, causing changes in photosynthetic efficiency and respiration. Effects also induce oxidative stress through ROS production and alter plant morpho-physiological functions, which greatly reduces crop productivity [24,34,35].

### 2.5. Soil Salinity Stress Effects in Crops

In contrast, several authors have indicated that salinity is one of the main types of abiotic stress that negatively affects plant growth and development, ultimately leading to a reduction in crop production [23,24,32,36,37]. According to Afridi et al. [36] approximately 800 million hectares of arable land around the world are affected by salinity, mainly in arid and semi-arid regions. This abiotic stress is analyzed in greater detail, as follows:

## 3. Soil Salinity’s Impact on Agricultural Production

Soil salinity refers to the presence of high concentrations of salts in soil, which harms plants due to their toxic effects and decreased soil osmotic potential. Technically, it is the concentration of all soluble salts in soil, translated as electrical conductivity. The most common method is to find soils with high levels of NaCl; however, they usually present different salt combinations such as Na^+^, HCO_3_^−^, Mg^+^, SO_4_^−^, K^+^, Cl^−^, Ca^+^, and CO_3_^−^ [12,38].

The constant rise in salinization of soil has caused it to be less productive, which encourages producers to invest more in agricultural inputs to obtain higher yields or to maintain the same production levels. Undoubtedly, these actions increase production costs, generating a domino effect across the entire production chain, which is more accentuated in developing countries [39]. Similarly, the loss of crops due to increased soil salinity affects the migration patterns of farmland, increases the agroforestry barrier, and negatively impacts the environment [40].

In this way, soil salinity is one of the most damaging abiotic processes for worldwide agriculture because it hinders plant growth and development, reduces crop yields, and suppresses growth and soil microbiota diversity [36,41,42]. Specific damages include delayed seed germination, reduction in root length, photosynthetic apparatus paralysis, and homeostatic processes such as water absorption, transport, and transpiration. Increased Na^+^ and Cl^−^ concentrations lead to the formation of ion imbalances in plants, resulting in reduced nutrient absorption, enzyme inactivation, protein synthesis inhibition, slow photosynthesis rate, and burning of leaves and stems [11,24]. In addition, salinity degrades the soil structure because it reduces porosity and water permeability [36].

**Figure 1:**
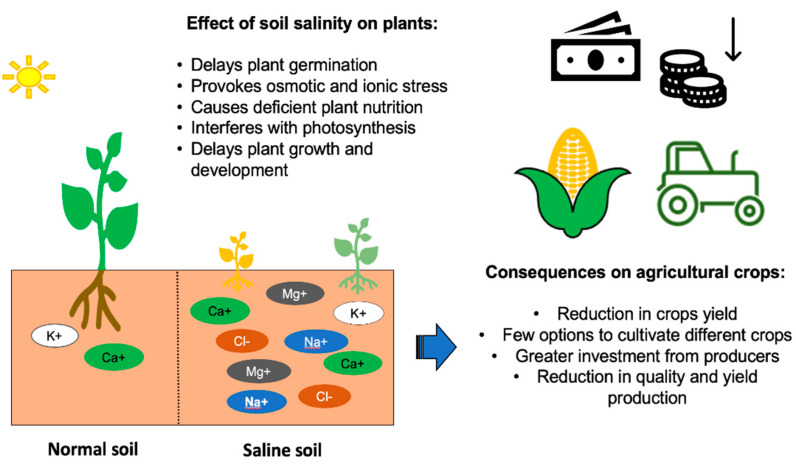
Effect of salts excess on plants and agricultural crops. Horizontal arrow: consequences of saline soils on agricultural crops. Vertical arrow: reduction of money gain.

In the 1990s, it was estimated that the proportion of soils affected by salinity worldwide was approximately 10% and that between 25% and 50% of irrigated areas were salinized. Currently, it is estimated that approximately 800 million hectares of arable land are affected by salinity. In addition, 32 million hectares are no longer arable, because of high salinity [43,44]. Impacts of this stress on the agricultural sector are alarming because it compromises food sovereignty and food security (Figure 1) [45].

## 4. Factors That Cause Gradual Increase in Soil Salinity

Salinity is the second cause of soil degradation and is therefore one of the factors that caused the decline of agricultural societies by 10,000 years [46]. At present, the exact extent of soil affected by salinity is not known; however, it is estimated that, annually, around 2000 hectares of arable land is lost worldwide due to this process, and this figure is expected to increase in the face of climate change, mainly in arid and semi-arid regions. The above is a result of a lack of environmental awareness, the irrational use of synthetic chemical fertilizers and water resources, in addition to other edaphoclimatic factors [6,46,47,48].

Salinity problems occur in all climatic conditions and are the result of natural and anthropic actions [6]. The first is given by mineral elements that contribute to soil salinity, such as underground brines, low precipitation, high rates of evaporation, proximity to mines, climate change, weathering, and rises in sea level due to global warming [46,47,48,49]. Anthropic causes are the result of deficient agricultural practices, such as the irrational use of chemical fertilizers and biological fertilizers (compost), and the incorrect management of irrigation water (deep wells), which allows salt mobility within soil and its transport to new sites, and solid and organic urban waste that contains enormous amounts of salts [41,42,46,48,49].

Minerals responsible for salinity problems in soil originate from sources that gradually accumulate in the soil [50]. In arid and semi-arid regions, low precipitation, rising groundwater levels, evapotranspiration, and low soil lixiviation are the main natural causes of increased salinity [48,51]. For example, in coastal areas, an increase in salinity has been observed as 1.4 times higher together with high levels of seawater intrusion, and a notable increase of 1.6 times in groundwater salinity has been reported during the last two decades [6,7,47]. In addition, it is anticipated that if the groundwater rises due to poor drainage and deep-rooted vegetation is replaced by shallow-rooted crops, groundwater will dissolve salts embedded in rocks and saltwater will rise to the surface and evaporate, causing further increases in soil salinity [48,52].

Similarly, climate change, caused by increases in greenhouse gas emissions, exerts strong pressure on soils [53]. In areas with shallow groundwater and fine-textured soils that are subject to intense rains or prolonged drought conditions, salinization is expected to increase, as well as in future climate change scenarios due to rising sea levels and temperatures [7,47,48]. For example, in the San Joaquin Valley, California, United States of America (USA), changes in precipitation patterns have caused a significant increase in soil salinity due to prolonged droughts. This contrasts with reports of the Red River Valley in Minnesota, USA, where extreme rain has contributed to an elevation in groundwater and soil salinization [47].

On the other hand, anthropogenic causes that have the greatest impact on soil salinization are the incorrect management of irrigation water and deficient agricultural practices [46,51,54,55]. It has been estimated that, globally, approximately 24% of irrigated lands are damaged by salinity, particularly where irrigation is carried out with low-quality groundwater, or by mixing seawater with freshwater [46,55]. Therefore, managing water quality should be an important component of irrigated agriculture, as these areas are prone to developing salinity [46,53,55].

Irrigation water quality varies in different countries and regions depending on how groundwater is extracted and used, as well as the intensity of rainfall and subsequent recharge of the aquifer. The use of seawater and groundwater for agriculture in regions with low rainfall leads to a surged salinity of groundwater and soil, limiting the selection of crops for agriculture [51,52,55]. For example, the application of saline water for agricultural irrigation has been associated with the development of salinity, sodicity, ionic toxicity, and soil contamination [53]. Machekposhti et al. [55] observed that the irrigation of sunflower plants with a mixture of seawater and fresh water leads to a significant increase in soil salinity, mainly when >30% seawater is used in the mixture.

Likewise, in semi-arid areas, fertilization practices favor salt concentration in soil; for example, fertilization with potassium in KCl form is the main salinization source in different parts of the world. In this sense, the contribution of Cl^−^ to groundwater salinization can vary depending on the intensity of evapotranspiration and agricultural practices [56].

## 5. Impact of Soil Salinity on Microbial Diversity and Plant Physiology

Soil salinity is a threat to global agricultural production and ecosystems because it changes soil characteristics, reduces plant growth, and affects microbial diversity and metabolism [57,58,59]. Saline soils are characterized by being formed under the influence of various salts, with different cations and anions in their solid or liquid phases, which actively affect soil structure, development, physical, chemical, and biological characteristics, as well as fertility [48,60].

The high sodium ion content in soil causes clay dispersion and organic matter that settles on surfaces of soil particles, which cover matrix spaces, block water infiltration, and reduce permeability [50,60]. Consequently, soils tend to present a loss of fertility due to flooding for extended periods of time after rain or irrigation, and to water erosion [50]. Simultaneously, waterlogging causes the separation and breakdown of soil aggregates due to wetting [48]; this, together with dispersion and clay expansion, modifies the original soil structure, which is considered the most important soil physical property related to plant growth [48,60].

Various reports confirm the above-mentioned; for example, in saline soils of the Nile Delta, where *T. aestivum* is grown, salinity increases electrical conductivity and bulk density, but causes decreases in organic matter, water availability, and hydraulic conductivity [61]. In addition, at salt extraction sites in Nigeria, salinity affected chemical properties, such as organic carbon and magnesium levels, as well as total nitrogen and phosphorous, which decreased significantly. However, the pH, electrical conductivity, exchangeable sodium percentage, and sodium absorption ratio increased [62].

Alternatively, soil microorganisms are involved in fundamental activities that ensure the stability and productivity of natural and agricultural ecosystems, as they facilitate the uptake of nutrients, produce phytohormones, decompose toxic substances, and improve soil structure [2,10,14,59]. Microbial diversity is influenced by biotic and abiotic factors; among the latter, salinity is one of the most relevant [59,63]. It has been shown that a high accumulation of salts in soil negatively affects microbial diversity and biomass, and changes the rhizosphere microbial community structure [10,57,64].

Rath et al. [57] and Jun-Yu et al. [64] found that phosphate-solubilizing, -ammonifying, -nitrifying, and -denitrifying bacterial abundance decreased with increasing salinity in soils cultivated with cotton (*Gossypium hirsutum*). Likewise, the salt content, percentage of exchangeable sodium, and pH were negatively correlated with enzymatic activities (amylase, catalase, urease, and alkaline phosphatase), biomass, and microbial respiration in agricultural soils. In soils of the hypersaline lake Ejinur in China, multiple regression and redundancy analyses indicated that bacteria and fungi were more affected by SO_4_^−^ and HCO_3_^−^, respectively [10]. Likewise, in *O. sativa* crops, the bacterial community composition changed with increasing salinity and correlated with the tolerance to salt and pH. In addition, the diversity decreased along salinity gradients, as well as with decreasing pH [57,63].

As mentioned above, salinity causes a loss of soil structure and promotes waterlogging, which prevents seedling emergence and delays root development [48]. In addition, salts retain water in soil with a high osmotic potential that limits the exchange of water and nutrients with plant roots; consequently, they retard plant growth and development [50,59]. Similarly, soil salinity restricts crop yield by affecting various physiological, biochemical, and molecular functions [8,65]. Salinity hinders plant germination, growth, photosynthesis, respiration, and stomatal conductance [65]. Furthermore, it reduces the water potential of leaves and turgor pressure and generates osmotic stress [65,66]. In contrast, the ROS content in plant cells increases because of toxicity and ion homeostasis alteration [66,67]. Thus, nutrient uptake is unbalanced and membrane disintegration takes place, involving certain ultrastructures and leading to osmotic and ionic stress [65].

## 6. Soil Microbial Diversity as a Source of Plant Growth-Promoting Bacteria (PGPB) Detection

Soil represents an ecosystem in which great microbial diversity can be detected. Some data indicate that up to 10 billion microorganisms can be found, which can correspond to thousands of different species. In fact, the observation of soil samples using epifluorescence micrograph after staining with 4, 6-diamidino-2-phenylindole indicated approximately 4.2 × 10^10^ cell gram^−1^ soil; when trying to recover by microbial culture, less than 1% of the soil sample was recovered (4.2 × 10^6^ colony-forming units g^−1^ soil) [68]. Even though only a percentage of cells or microbial species can be recovered through plating, soil is an inexhaustible source of microorganisms that play fundamental roles in biogeochemical cycles and soil fertility [69,70].

Soil, in addition to being biodiverse, represents a complex ecosystem, with a great variety of environmental factors that modulate microbial communities, including PGPB. Some studies have shown that the diversity and structure of microbial communities can vary depending on space and time, as well as factors such as pH, temperature, soil type, geography (altitude and latitude), and climatic conditions (availability of water and UV radiation) [71]. These environmental conditions modulate microdiversity in bulk and rhizosphere soils, with indirect effects on plant health and growth [72,73].

Some massive DNA sequencing techniques help to understand the association between certain factors and the structure of soil microbial communities. Roesch et al. [74] evaluated microbial diversity in four soil types across a large transect of the western hemisphere, including three agricultural soils from a *Z. mays* field in Rio Grande do Sul, Brazil, a sugarcane (*Saccharum officinarum*) field in the Everglades Agricultural Area in Florida, and in soil from the Morrow Plots at the University of Illinois in Urbana, USA. A fourth soil sample was collected from a boreal forest site in northwestern Ontario, Canada. The results of this study allowed us to conclude that the most abundant bacterial groups in all four soils were Bacteroidetes, Betaproteobacteria, and Alphaproteobacteria, using three estimators of diversity. Additionally, results revealed that the bacterial diversity of forest soil was phylum-rich compared to that of the agricultural soils, which were species-rich but poor at the phylum level. In conclusion, the authors demonstrated that the agricultural management of soil may significantly influence the diversity of bacteria and other microbial groups, such as archaea.

In this sense, agricultural ecosystems represent important sources for the isolation and selection of beneficial bacteria, with wide potential to produce metabolites and compounds with active ecological roles [75]. Some PGPB belonging to the Proteobacteria class, mainly Alphaproteobacteria (*Rhizobium*, *Sinorhizobium*, *Ensifer*, *Bradyrhizobium*, and *Mesorhizobium*), Betaproteobacteria (*Nitrosomonas*, *Burkholderia*, *Paraburkholderia*, and *Cupriavidus*), Gammaproteobacteria (*Azotobacter* and *Pseudomonas*), Firmicutes (*Bacillus*, *Peanibacillus*, and *Neobacillus*), and Actinobacteria (*Arthrobacter*, *Actinomyces*, *Micrococcus*, and *Streptomyces*), are among the most common inhabitants of bulk and rhizosphere soils [76].

In a recent study, Sheirdil et al. [77] isolated ten strains of bacteria from sandy loam soil and observed that they have great potential to promote the growth and production of *T. aestivum* plants. Sequencing of the 16S rRNA gene identified two main genera of beneficial strains, *Bacillus* and *Pseudomonas*. These two genera are among the most widely documented PGPB in the literature, whose mechanisms of plant growth promotion and pathogen biocontrol include those with direct action, such as hormone production and nutrient facilitation, or indirect action, mainly by controlling phytopathogen attack. Likewise, the isolation of beneficial bacteria can be achieved from both rhizospheric and nonrhizospheric soils (bulk soil). For example, Pathak et al. [78] isolated free-living bacteria, mainly *Bacillus* and *Pseudomonas*, with good characteristics in promoting potato (*Solanum tuberosum*) growth and production, in addition to exhibiting antifungal activity against *Pythium* sp. and *Fusarium* sp. Within the screening of beneficial mechanisms, activities include the production of indole acetic acid (IAA), ammonia, hydrogen cyanide (HCN), siderophore presence, and phosphate solubilization (P-solubilization).

The isolation of native PGPB and their subsequent inoculation into the same ecosystems from where they were isolated is a relevant strategy that can reduce the possible lack of adaptation to environmental and biotic conditions of local soils. This method has been implemented in various studies, where consistent results have been observed in terms of improvements to soil fertility and whose actions enhance the intake of nutrients and elements such as P and N, particularly in calcareous soils, as shown by the work of Fan et al. [79]. Another example of the use of native strains was recently reported by Aynalem et al. [80], who isolated strains of *Bacillus thuringiensis* to control diseases caused by *Tuta absoluta* (tomato leafminer), which is one of the main agricultural pests that attack *S. lycopersicum*.

The above examples represent excellent efforts to prepare PGPB isolated from different types of soil, including those that are influenced by plant root exudates. However, the enormous abundance of strains of the genera *Bacillus* and *Pseudomonas* (among a few other genera) dominate the list of PGPB as biofertilizers or biopesticides [81]. Therefore, greater isolation and screening efforts are required to detect proposed new species that allow the expansion of options to use effective bioinoculants in various types of soils [82], where edaphoclimatic characteristics are variable and sometimes highly changeable according to the time of year.

## 7. Mechanisms of Tolerance to Saline Stress by PGPB

Soil is the main reservoir for bacteria that interact with plants and has been described as the most diverse ecosystem on earth. The soil microbiome is responsible for many biological processes that affect plant development. According to the above-mentioned, in agricultural crops, bacteria can modulate production by assisting and controlling nutrient acquisition and promoting stress tolerance [83]. Among salinity-tolerant genera and plant growth promoters (2–25%) that have been most frequently described are *Arthrobacter*, *Alcaligenes*, *Pseudomonas*, *Bukholderia*, *Bacillus*, *Flavobacterium*, and *Rhizobium*, which have been reported to decrease effects of salt stress in several crops [84,85].

**Figure 2:**
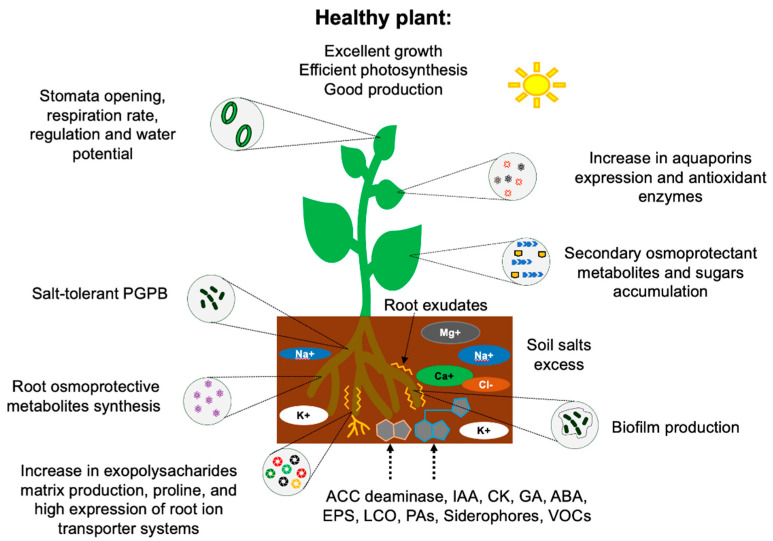
Salt tolerance mechanisms promoted by plant growth-promoting bacteria (PGPB). After that, PGPB colonize the surface of plant roots growing in saline soil, and a plant–bacteria communication is established through the synthesis of chemical signals that improve the plants’ physiology, growth, and reproduction. ACC deaminase: 1-aminociclopropane-1-carboxylase deaminase enzyme. IAA: Indole acetic acid. CK: Cytokinins. GA: Gibberellins. ABA: Abscisic acid. EPS: Exopolysaccharides. LCO: Lipo-chitooligosaccharides. PAs: Polyamines. VOCs: Volatile organic compounds.

Although interactions involving salinity-tolerant bacteria and plants are under investigation, recent reports indicate that the tolerance to salt promoted by PGPB can be at three levels: (a) Survival of the bacterium itself. (b) Induction of salt stress tolerance events in plants. (c) Improvement in soil quality [84]. Salinity-tolerant rhizobacteria have developed several mechanisms that allow them to survive under these conditions, which, in general, can be grouped into four main categories: (a) Osmotic balance. (b) Ionic homeostasis. (c) Signaling by phytohormones and production of extracellular molecules. (d) Nutrient acquisition (Figure 2). These mechanisms are summarized in Table 1 and are briefly described below, as they induce several physiological, morphological, and molecular changes that culminate in an induced systemic tolerance in plants. For the above-mentioned, PGPB improve morphological traits such as germination, seedling vigor index, roots and shoots lengths, and the fresh and dry biomass of stressed plants [86].

### 7.1. Osmotic Balance

Water is essential in biological processes, so the accumulation of salt alters the water uptake in plant cells, producing osmotic stress and ionic toxicity that affect development and growth [120]. In this context, it has been observed that PGPB regulate water potential and stomatal opening by modulating and improving the conduction of water and the rate of transpiration. For example, in *Z. mays* plants under saline stress inoculated with *B. megaterium*, water conductivity was improved by the induction of *ZmPIP1;1* and *ZmPIP1;5* gene expression, which encode for aquaporins [91]. Aquaporins are integral membrane proteins that facilitate water transport and other solutes between cells [121].

In addition, to counteract the effects of salinity, PGPB induce the production of osmoprotective metabolites, which maintain turgor pressure and ionic flux through the membrane. A wide range of secondary metabolites have been reported as compatible osmoprotectants/solutes, alcohols, glucosyl glycerol, betaines, amino acids, and tetrahydropyrimidine, which play a crucial role in improving salinity stress in plants. These metabolites also help to buffer saline stress faster, thereby improving the productivity of saline soil [120,122].

In another study by Jha et al. [123], *O. sativa* plants were cultivated under saline stress and inoculated with the bacterium *P. pseudoalcaligenes*, observing that the accumulation of glycine betaine-like compounds was stimulated, which improved stress tolerance in the crop. Another study reported that *Z. mays* plants inoculated with *Bacillus* HL3RS14 showed better development under saline stress, exhibiting high levels of proline, glycine, betaine, and malondialdehyde (MDA) in inoculated versus control plants [124]. Similarly, it was observed that seeds of *T. aestivum* inoculated with *B. aquimaris* increased the accumulation of sugars, which were growing under saline stress. Such bioinoculation stimulates the growth of plants [125].

### 7.2. Ionic Homeostasis

Under salt stress conditions, PGPB limit plant ion uptake by matrix production (exopolysaccharides), altering the root structure with numerous rhizosheaths, and regulating the expression of high-ion-affinity transporters [126]. In accordance with this, it has been reported that *G. max* plants inoculated with halotolerant *P. pseudoalcaligenes* (SMR-16) and *B. subtilis* (SMR-3) strains induced salt stress tolerance when plants were exposed to salinity (100 mM), as both activated 1-aminocyclopropane-1-carboxylate (ACC) deaminase activity, siderophore, and indole acetic acid (IAA) production. In addition, *P. pseudoalcaligenes* inoculated with *G. max* plants showed tolerance to increased protection activities, such as ion transport systems that reduced Na+ concentration, antioxidant enzymes, and proline, as well as a reduction in MDA production in both shoots and roots [127]. In another study, it was found that *P. koreensis* AK1 reduced Na^+^ concentrations and raised K^+^ in *G. max*, both in the leaves and roots [128].

In addition, in *Z. mays*, it has been reported in separate studies that *Serratia liquefaciens* KM4 and *Bacillus* sp. improved their growth under saline stress, modulating several physiological processes, including ionic homeostasis, by decreasing the uptake of Na^+^ and increasing that of K^+^ [103,129]. In addition, it has been documented that bacteria, in addition to helping maintain ionic balance, also possess other characteristics that contribute to the tolerance to salt stress [116].

### 7.3. Signaling by Phytohormones and Extracellular Molecules

Growth regulators or phytohormones are molecules that affect plant growth and development at low concentrations. The capacity of several bacteria to promote plant growth and development of the root system is one of the parameters used to determine the effectiveness of PGPB. These bacteria modulate several signaling pathways through phytohormone production, which contributes to salinity stress tolerance. In addition, plant–bacteria interactions stimulate plant phytohormone production. Those most frequently produced by PGPB are auxins (IAA mainly), cytokinins (CK), gibberellins (GA), abscisic acid (ABA), volatile organic compounds (VOCs), and ethylene (E). However, in E, its synthesis is reduced by ACC deaminase activity [130]. During salt stress, PGPB, which produce or stimulate the production of phytohormones, promote stress tolerance in plants. For example, IAA produced by PGPB is currently the most widely studied bacterial signaling molecule, which is synthesized in various ways, one of which is from tryptophan present in root exudates that is later transformed into IAA, which is absorbed by the plants. Thus, this molecule stimulates cell growth and proliferation as well as lateral root development, which increases the plant’s access to soil nutrients [126]. Recently, it was reported that IAA produced by *Leclercia adecarboxylata* M01—a PGPB—is related to sugar synthesis, organic acid production, and chlorophyll fluorescence improvement in *S. lycopersicum* [111]. Therefore, IAA is the main auxin that promotes plant growth [12,131].

In contrast, CK participates in potential cell maintenance in root and shoot meristems [118]. In addition, increased plant growth is related to the CK produced by PGPB, according to Arkhipova et al. [132]. Furthermore, López-Bucio et al. [133] reported that CK receptors in *B. megaterium* UMCV1 play an important role in plant growth promotion in *Arabidopsis thaliana* and *P. vulgaris*. In relation to GA, very few PGPB are known to produce these phytohormones. However, its importance has been reported since the 1980s. For example, Bottini et al. [134] reported that the increase in growth and yield of several crops is due to the production of GAs (GA1, GA3, and iso-GA3) by endophytic bacteria, such as *Azospirillum lipoferum*. In addition, Yanni (2001) indicated that *Rhizobium* strains produce GA7. More recently, Kang et al. [135] reported that the isolation and characterization of a new strain of *Leifsonia soli* sp. SE134 produces 11 GAs (GA1, GA4, GA7, GA8, GA9, GA12, GA19, GA20, GA24, GA34, and GA35). Experiments on plant growth in cucumber, tomato, and radish showed the great potential of this strain as a PGPB.

The regulatory roles of the ABA signaling pathway go well beyond stomatal movement and seed dormancy [136]. For instance, ABA is best known for its function as a stress-related metabolite, is ubiquitous throughout the plant kingdom, and participates in the accumulation of osmolytes, as well as the levels of Ca^+^ and K^+^ [137,138]. ABA is also synthesized by PGPB strains, including *B. licheniformis* and *P. fluorescens* [139]. Lastly, the inoculation of *Chrysanthemum* plants with *B. licheniformis* SA03 ameliorated the detrimental effects of saline–alkaline stress by improving ABA levels, which confirms that SA03 helps host plants tolerate saline–alkaline stress [140].

Finally, a gaseous phytohormone that accumulates during stress is E. Under normal conditions, E participates in germination, growth, root hair elongation, and fruit ripening, although under stress conditions, it inhibits plant growth. PGPB modulate the level of E through ACC deaminase production, which transforms the precursor of E synthesis, ACC, into ammonium and alpha-ketobutyrate [130]. Halotolerant bacteria that can promote plant growth through the production of ACC deaminase include *Arthrobacter*, *Bacillus*, *Brevibacterium*, *Gracilibacillus*, *Virgibacillus*, *Salinicoccus*, *Pseudomonas*, and *Exiguobacterium* [85].

Under saline stress conditions, PGPB produce extracellular molecules that positively impact plant development, defensive functions, growth stimulation through the induction of defense against pathogens, and stress tolerance [126]. Of these molecules, the most studied are exopolysaccharides (EPS), which stabilize the structure of the soil and increase water availability and ion exchange. In addition, they are important in biofilm formation, which confers resistance to bacteria against adverse environmental conditions [141].

Other extracellular compounds produced by PGPB are lipo-chitooligosaccharides (LCO) synthesized by rhizobia that participate in nodulation processes [142]. Mention may also be made of bacteriocins, small antimicrobial peptides produced by several rhizobacteria with antibacterial activity on other competing bacteria; it has been described that they can help plants to counteract salt stress too. Polyamines (PAs) are small aliphatic nitrogenous molecules with antioxidant activity that stimulate stress tolerance in plants. VOCs are organic molecules released by PGPB that stimulate plant growth and contribute by reducing the adverse effects of salinity stress. Finally, PGPB can produce antioxidant enzymes, such as peroxidase, superoxide dismutase, polyphenol oxidase, and catalase, which scavenge excess ROS generated during saline stress in plants [36,126].

### 7.4. Nutrient Uptake

Plant nutrition has attracted the attention of researchers around the world because it is directly related to crop yield and quality, especially under stress conditions. Salinity affects water transport and nutrients to roots; for instance, low levels of nitrogen, phosphorus, potassium, and other micronutrients are some of the reasons for poor plant growth. Due to salinity, the uptake of nutrients by vegetable roots is affected [143].

Among the most widely studied mechanisms through which PGPB improve nutrient uptake is nitrogen fixation, an activity widely distributed in rhizospheric bacteria. Among the bacterial genera capable of fixing atmospheric nitrogen when they colonize plants, other than legumes, genera, such as *Azotobacter*, *Azospirillum*, *Pseudomonas*, *Agrobacterium*, *Erwinia*, *Bacillus*, *Serratia*, *Klebsiella*, and *Burkholderia*, have been suggested to improve plant growth under conditions of salinity stress [143]. In addition to nitrogen fixation, these bacteria can use other strategies that contribute to tolerance to salinity stress [4].

Phosphorus is an essential element for plant development and can be found in organic and inorganic molecules; however, in saline soil, it is common for it to be found in the form of insoluble compounds. Several halotolerant bacteria can solubilize phosphate through chelation, ion exchange, or soil acidification [85]. The inoculation of cultures with halotolerant and phosphate-solubilizing bacteria belonging to the genera *Arthrobacter*, *Bacillus*, *Beijerinckia*, *Burkholderia*, *Enterobacter*, *Pseudomonas*, *Erwinia*, *Mesorhizobium*, *Flavobacterium*, *Rhorandococcus*, and *Klebsiella* resulted in the reduction in the adverse effects of salinity stress [85,143].

### 7.5. Other Roles of PGPB as Alternatives to Ameliorate Saline Stress in Plants

The potential for plant growth promotion under conditions of salt stress by halotolerant bacteria has been described for various genera, such as *Arthrobacter*, *Azospirillum*, *Alcaligenes Bacillus*, *Burkholderia*, *Enterobacter*, *Flavobacterium*, *Pseudomonas*, and *Rhizobium*. When these bacteria are applied as bioinoculants, they also improve some soil properties, such as organic matter content, soil structure, and water retention [84]. Therefore, given the estimates that climate change will enhance the severity of salinity, drought, and frost—among others—it is imperative that the exploration of beneficial microbiota be increased to promote sustainable agriculture, environmental protection, and food safety [126].

In accordance with the above, the potential of PGPB has been demonstrated in studies carried out under conditions of saline stress, where it was determined that the inoculation of these bacteria improved crop performance. It has been described that when a variety of *T. aestivum* susceptible to salinity was grown under saline stress and inoculated with *P. fluorescens*, several crop yield parameters were improved: number of spikes (76.6%), length of spikes (85.9%), and weight of 100 grains (32.9%) [144]. Similarly, Rajput et al. [145] found that *Planococcus rifietoensis* improves the salinity tolerance of *T. aestivum* and increased the yield (weight of 100 grains) from 5.7 to 12% in field experiments.

## 8. Soil Salinity Problem in Mexico: A Case Study

Soil degradation includes a series of physical, chemical, and biological changes that lead to the deterioration of soil quality. Chemical degradation is very common and is characterized by reduced fertility, acidification, contamination, eutrophication, and salinization/alkalization. According to official reports, it is estimated that in Mexico, 17.8% (34 million hectares) of the national territory is associated with some degree of affectation due to soil chemical degradation. In addition, it is estimated that in 3.2% of the soils (representing approximately 1 million hectares), the chemical degradation of soils can be attributed to salinity/alkalinity [146].

In Mexico, saline soils are located mainly in the arid and semi-arid irrigated areas of the center and north of the country, as well as along the coasts. The main states that exhibit soil chemical degradation in response to salinity are also those that have the highest participation in agricultural production (Sinaloa, Guanajuato, Tamaulipas, Sonora, San Luis Potosí, Chiapas, Nuevo León, Oaxaca, Veracruz, Zacatecas, and Michoacán) [147,148].

In accordance with the above, the noncoastal saline areas of Mexico are mostly home to soils with irrigated agricultural activity, which is due to the use of low-quality water, in addition to the excessive use of water in soils with poor or nonexistent drainage systems. Moreover, one-third of the water used for agricultural irrigation—on a nationwide scale—comes from aquifers; a significant percentage of this water has been overexploited and is characterized by the presence of high levels of soluble salts. The most productive agricultural areas of the country are organized into irrigation districts, which account for approximately 3.4 million hectares, and approximately 30% of these districts have salinity problems [149].

The irrigation district 24 of La Ciénega de Chapala is in the western part of Michoacán state and covers 11,520 irrigation hectares. In this area, agricultural production is continuously affected by soil salinization. For example, studies carried out in this area indicate that salinity ranges from very low to severe, with electrical conductivity (EC) values between 1.5 and 40 dS/m [150,151]. Increasing salinity has been reported to be mainly due to the water salts used for irrigation. Accordingly, 81% of the water for agricultural use in district 24 comes from surface sources and the remaining 19% comes from groundwater. It has been observed that two sources present salinity; in the case of surface water, it presents an EC between 0.4 and 1.25 dS/m, while that of subsoil is between 0.5 and 4.5 dS/m. Taking into consideration these data and characteristics of soil in the area, it has been considered that due to its salinity, water for agricultural use in irrigation districts is associated with a moderate to high risk of soil salinization [148,150]. Below, we suggest some strategies that provide a solution to the problem of soil salinization and that include the application of PGPB for a better and sustainable agricultural production.

## 9. Future Recommendations

Due to the magnitude of the problem at the global, national, and regional levels, the recovery of saline soil is important for agricultural activities, and therefore, for food security. There are different ways to deal with the problem of soil salinity; one consists of remediating saline soils using physical, chemical, or biological methods, although many of the methods applied to large areas of soil are not economically viable [152]. A second alternative consists of its previous remediation, using species of crops resistant to salinity or susceptible crops, but inoculated with PGPB that improve tolerance to salinity through several strategies [4]. However, many crops of food interest are susceptible to salinity; therefore, the use of PGPB, which enhances the tolerance to salts in crops, has been seen as the most promising alternative.

The use and application of PGPB to ameliorate the harmful effects of soil salinity, among other types of abiotic stress, is also an eco-friendly option, as recent studies have shown that they are an alternative to reduce the application of agrochemicals, which generate constant pollution to the environment [76]. In recent years, the mentality of Mexican agricultural producers has undergone a change, in part due to restrictions on the export and import of products in countries such as USA, Canada, and Germany, which have chosen to market and add value to the systems of organic production. In this way, it is highly suggested to re-educate the population and agronomists to improve their production systems with other types of production without agrochemicals [76].

Executing prophylactic systems with bioinoculants in seeds before sowing would be a future control of possible infections by pathogens. Various studies have shown that such inoculation in plants such as maize, sorghum, or wheat have resulted in better interactions with the plant, increasing its production and protection against pathogens [152,153,154,155]. However, this would require, again, the re-education of production forms, particularly in developing countries [156].

Recently, Mukhopadhyay et al. [41] published a review article where they explored the effect of climate change on soil salinity, a topic of particular interest to the vast majority of world regions, as, although it is expected that there will be more affected regions in the next 50 years, no one will be exempt from its effects. Therefore, new agricultural practices are required that consider these changes in the next 5 or 10 years, in order to have arable soils that maintain their efficient production. Some suggestions are the reduction in the use of chemical fertilizers that generate a greater salinization of the soil, avoid evapotranspiration, as well as the addition of waters containing a high content of salts, among other suggestions, such as the application and greater use of bioinoculants containing PGPB [156]. 

## 10. Conclusions

The production of safe and sufficient food represents one of the greatest challenges for all countries in the world in the coming decades due to the increase in the world population, climate change, and the impact of abiotic stress on crop productivity. Soil salinity stress is one of the most important abiotic factors that significantly reduces global and local agricultural production. To address this problem, several strategies have been proposed, among which the use of PGPB is highlighted, as this is a sustainable alternative that improves saline soil conditions and simultaneously increases the plant production, which could reduce the use of synthetic chemical fertilizers [157]. The mechanisms by which PGPB induce physiological, morphological, and molecular changes in plants rely on osmotic balance, ionic homeostasis, phytohormone signaling, extracellular molecule production, and nutrient acquisition, ultimately alleviating salinity stress and increasing production. These mechanisms represent an excellent opportunity to select the best PGPB, which ensures successful results in the field, where soils are also important as an inexhaustible source of beneficial microorganisms.

## Figures and Tables

**Table 1:** Plant growth-promoting bacteria (PGPB) and their mechanisms targeted at helping plants to deal with salinity stress.

PGPB Species	Mechanisms	Reference
*Rhizobium* spp., *Bacillus megaterium*, *Pantoea agglomerans*, *Azospirillum* sp., *Pseudomonas mendocina*	Aquaporins	[87,88,89,90,91,92,93]
*Brevibacterium iodinum* KNUC7183, *Rhizobium massiliae* KNUC7586, *Bacillus* sp., *Arthrobacter pascens*, *Bacillus firmus* SW5, *Bacillus fortis*	Sugars, and proline, chlorophyll synthesis, nutrient uptake, gas exchange parameters, osmolytes levels, total phenolic and flavonoid contents, and antioxidant enzymes activities	[94,95,96,97,98,99,100,101,102,103,104,105,106,107,108,109,110]
*Leclercia adecarboxylata* M01, *Pseudomonas* sp. UW4, *Arthrobacter woluwensis* AK1, *Microbacterium oxydans* AK2, *Arthrobacter aurescens* AK3, *Bacillus megaterium* AK4, *Bacillus aryabhattai* AK5, *Pseudomonas* sp. UW4	ACC deaminase production, trehalose, IAA, siderophores, and GA. Increased phosphate solubilization	[111,112,113]
*Bacillus siamensis* PM13, *Bacillus* sp. PM15, *Bacillus methylotrophicus* PM19, *Citrobacter freundii* ATHM38, *Bacillus aryabhattai*, *Achromobacter denitrificans*, and *Ochrobactrum intermedium*	Exhibited atmospheric nitrogen fixation, phosphate solubilization, IAA, enhanced EPS production, and ACC deaminase	[114,115,116]
*Bacillus amyloliquefaciens* FZB42, *Bacillus subtilis* GB03	Increase in VOCs synthesis, peroxidase, catalase, and superoxide dismutase, as well as in sugar total content. Decreased Na^+^ content, homeostasis, *increased chlorophyll,* antioxidant enzymes	[117,118,119]

ACC deaminase: 1-aminociclopropane-1-carboxylase deaminase enzyme. IAA: Indole acetic acid. GA: Gibberellins. EPS: Exopolysaccharides. VOCs: Volatile organic compounds.
